# Differential regulation of cellular stress responses by the endoplasmic reticulum‐resident Selenoprotein S (Seps1) in proliferating myoblasts versus myotubes

**DOI:** 10.14814/phy2.13926

**Published:** 2018-12-17

**Authors:** Alex B. Addinsall, Sheree D. Martin, Fiona Collier, Xavier A. Conlan, Victoria C. Foletta, Nicole Stupka

**Affiliations:** ^1^ Centre for Molecular and Medical Research School of Medicine Deakin University Geelong Australia; ^2^ GCEID, University Hospital Barwon Health Geelong Australia; ^3^ School of Medicine Deakin University Geelong Australia; ^4^ Centre for Chemistry and Biotechnology School of Life and Environmental Sciences Faculty of Science, Engineering and Built Environment Deakin University Geelong Australia; ^5^ Institute for Physical Activity and Nutrition (IPAN) School of Exercise and Nutrition Sciences Deakin University Geelong Australia

**Keywords:** ER stress, myoblast, myotube, oxidative stress, palmitate, Selenoprotein S

## Abstract

The antioxidant Selenoprotein S (Seps1, *Selenos*) is an endoplasmic reticulum (ER)‐resident protein associated with metabolic and inflammatory disease. While Seps1 is highly expressed in skeletal muscle, its mechanistic role as an antioxidant in skeletal muscle cells is not well characterized. In C2C12 myotubes treated with palmitate for 24 h, endogenous Seps1 protein expression was upregulated twofold. Two different siRNA constructs were used to investigate whether decreased levels of Seps1 exacerbated lipid‐induced oxidative and ER stress in C2C12 myotubes and myoblasts, which differ with regards to cell cycle state and metabolic phenotype. In myoblasts, Seps1 protein knockdown of ~50% or ~75% exacerbated cellular stress responses in the presence of palmitate; as indicated by decreased cell viability and proliferation, higher H_2_O_2_ levels, a lower reduced to oxidized glutathione (GSH:GSSG) ratio, and enhanced gene expression of ER and oxidative stress markers. Even in the absence of palmitate, Seps1 knockdown increased oxidative stress in myoblasts. Whereas, in myotubes in the presence of palmitate, a ~50% knockdown of Seps1 was associated with a trend toward a marginal (3‐5%) decrease in viability (*P *=* *0.05), decreased cellular ROS levels, and a reduced mRNA transcript abundance of the cellular stress marker thioredoxin inhibitory binding protein (*Txnip*). Furthermore, no enhancement of gene markers of ER stress was observed in palmitate‐treated myotubes in response to Seps1 knockdown. In conclusion, reduced Seps1 levels exacerbate nutrient‐induced cellular stress responses to a greater extent in glycolytic, proliferating myoblasts than in oxidative, differentiated myotubes, thus demonstrating the importance of cell phenotype to Seps1 function.

## Introduction

Selenium is an essential micronutrient (Brown and Arthur 2001). Cellular stress responses, metabolism and immunity are regulated by selenium in the form of selenocysteine (Sec), the 21st amino acid, which is incorporated into selenoproteins (Brown and Arthur 2001; Shchedrina et al. 2010; Li et al. 2014). There are 25 selenoproteins in the human genome (Kryukov et al. 2003), including seven endoplasmic reticulum (ER)‐resident selenoproteins (*DIO2, SELENOF, SELENOK, SELENOM, SELENON, SELENOS,* and *SELENOT*). The function of these selenoproteins is not well characterized; however, they have been implicated in oxidative and ER stress responses, inflammation, calcium regulation, and the maintenance of membrane‐associated multiprotein complexes ((Shchedrina et al. 2011; Turanov et al. 2014; Li et al. 2018); reviewed here (Liu and Rozovsky 2015; Addinsall et al. 2018a)).

Selenoprotein S (*SEPS1, SELENOS*) is an ER‐resident transmembrane protein. Its cytoplasmic region contains a coiled‐coil domain which includes an intrinsically disordered region important for protein binding (Turanov et al. 2014). The N terminus Sec residue is also localized to this cytoplasmic region and contributes to protein oxidoreductase activity (Liu and Rozovsky 2013; Liu et al. 2013). Seps1 is ubiquitously expressed with particularly high expression in metabolically active tissues including skeletal muscle, where it has been shown to have a role in regulating contractile function and inflammation (Wright et al. 2017; Addinsall et al. 2018b). Single nucleotide polymorphisms in the *SEPS1* gene have been linked to various human diseases associated with heightened metabolic and/or inflammatory stress (Sun et al. 2016) including cardiovascular and metabolic diseases (Curran et al. 2005; Alanne et al. 2007; Olsson et al. 2011).

In vitro*,* Seps1 is protective against oxidative stress and ER stress in a number of cell types. In response to stimulation by tunicamycin (an N‐glycosylation inhibitor) or thapsigargin (an inhibitor of ER Ca^2+^ ATPase activity), the activity of the ER stress response element (ERSE) in the Seps1 promoter region is increased (Gao et al. 2006). Seps1 is thought to be functionally active in the adaptive arm of the unfolded protein response (UPR). Specifically, ER‐associated protein degradation (ERAD), where Seps1 forms a multiprotein complex with selenoprotein K, derlin‐1, p97 ATPase and an E3 ubiquitin ligase at the ER membrane to facilitate the translocation of misfolded proteins to the cytosol for degradation by the ubiquitin proteasome pathway (Ye et al. 2004; Curran et al. 2005; Shchedrina et al. 2010; Christianson et al. 2012). As an antioxidant, Seps1 has oxidoreductase activity against H_2_O_2_ and is most effective when coupled with thioredoxin (Liu and Rozovsky 2013; Liu et al. 2013). This is interesting, given the regulation of various oxidative and inflammatory stress sensitive signaling pathways by the thioredoxin antioxidant system (Mahmood et al. 2013). Seps1 also has the potential to reduce disulfide bonds. This is likely relevant to its role in adaptive ER stress responses and the retrograde translocation of misfolded proteins out of the ER for cytosolic degradation (Ye et al. 2004). Furthermore, in a proteomics screen using HEK 293 cells, Seps1 was present in various multiportion complexes, and thus may have a broad and complex role in regulating stress sensitive signaling pathways beyond its well‐described role in the UPR (Turanov et al. 2014).

There is good in vitro evidence to support a role for Seps1 in regulating cellular stress responses; although, there is increasing evidence that the protective properties of Seps1 are dependent upon cell phenotype (Wright et al. 2017; Addinsall et al. 2018b). Seps1 overexpression in RAW 264.7 macrophages improved cell viability following the pharmacological induction of ER stress (Kim et al. 2007). Whilst Seps1 suppression increased cell death in RAW 264.7 macrophages (Kim et al. 2007), in primary astroglial cells (Fradejas et al. 2008), and in murine Hepa1‐6 (Li et al. 2018) and human HepG2 hepatoma cells (Du et al. 2010b). SEPS1 overexpression was also protective against oxidative stress in human umbilical vein endothelial cells (HUVECs) treated with H_2_O_2_ (Zhao et al. 2013). Accordingly, Seps1 gene knockdown was associated with increased reactive oxygen species (ROS), altered activity of primary antioxidant defense enzymes and reduced viability in HUVECs (Zhao et al. 2013), HepG2 cells (Zeng et al. 2008), and vascular smooth muscle cells (Ye et al. 2016).

In individuals with obesity, insulin resistance and type 2 diabetes, the excess accumulation of lipids, including saturated fatty acids in skeletal muscle, is associated with increased cellular stress (Anderson et al. 2009; Flamment et al. 2012). Whilst some selenoproteins are important for skeletal muscle health and function (reviewed in (Rederstorff et al. 2006)) and SEPS1 is associated with metabolic disease (Karlsson et al. 2004; Du et al. 2008; Yu et al. 2016) including circulating plasma triglyceride concentrations in vivo (Walder et al. 2002), its specific role in response to excess saturated fatty acids in skeletal muscle has to date not been described. Here, using two different siRNA constructs, Seps1 was knocked down in proliferating myoblasts and differentiated myotubes, which also differ with regards to their metabolic phenotype, to gain insight into whether Seps1 is protective against palmitate treatment in these different cellular states. Cell viability, cell cycle progression, ROS levels, and gene and protein markers of oxidative and ER stress were assessed.

## Materials and Methods

### Cell culture

Murine C2C12 myoblasts were cultured in 5% CO_2_ and atmospheric O_2_ at 37°C in growth media consisting of 5 mmol/L (low glucose) DMEM supplemented with 10% fetal bovine serum (FBS; In Vitro Technologies, Noble Park, AUS). To stimulate myotube formation, confluent myoblasts were cultured in differentiation media consisting of 5 mmol/L glucose DMEM supplemented with 2% horse serum (HS; Life Technologies, Mulgrave, AUS), refreshed every 24 h.

### Palmitate preparation

Palmitic acid (250 mmol/L; Sigma‐Aldrich, Castle Hill, AUS) was dissolved in 95% ethanol at 50–60°C, followed by dilution to 4.5 mmol/L in 5 mmol/L glucose DMEM (Life Technologies, Mulgrave, AUS) containing 20% fatty acid‐free bovine serum albumin (BSA; Sigma‐Aldrich). BSA and palmitate were conjugated at 45°C for 1 h with gentle mixing. Solutions were filter‐sterilized and diluted to the appropriate concentration in cell culture media. Control media (vehicle) contained ethanol and BSA in the absence of palmitate (Rakatzi et al. 2004). Myoblasts and myotubes were treated with 0.1 mmol/L and 0.35 mmol/L palmitate or vehicle for 21 h, respectively. Myotubes were exposed to an additional 3 h incubation with serum‐free media containing palmitate or vehicle after which the cells were collected for experimental analysis. The respective palmitate doses were optimized in pilot experiments to minimize toxic effects of palmitate exposure on cell viability (Lancaster et al. 2018), with 0.2 mmol/L to 0.75 mmol/L palmitate tested in myotubes and 0.05 mmol/L to 0.2 mmol/L palmitate tested in myoblasts (data not shown). Myoblasts were treated with lower doses of palmitate and not exposed to serum‐free palmitate or vehicle due to their greater sensitivity to lipid stress (Grabiec et al. 2015).

### Stealth™ siRNA transfection

The following Stealth™ RNAi constructs were used siRNA1 (5′‐GGAAGAUCUAAAUGCCCAAGUUGAA‐3′) and siRNA2 (5′‐CAUGCAAGAAGGCAGAAGUUACAAA‐3′) (Invitrogen, Mulgrave, AUS). For the myoblast experiments, C2C12 myoblasts were transfected at 40–50% confluence using the Stealth™ RNAi duplexes or a Stealth™ RNAi negative control duplex (Invitrogen) with a 40% G/C content and lipofectamine RNAiMAX (Invitrogen), according to manufacturer's instructions. The final concentration of both Stealth™ RNAi duplexes and the negative control was 10 nmol/L. Twenty‐four hours posttransfection, myoblasts were treated with palmitate or vehicle as described above. The timing of transfection and palmitate treatment was optimized such that myoblasts were still actively proliferating and subconfluent at end of the experiments. For the myotube experiments, to minimize the potentially confounding effects of palmitate (Grabiec et al. 2015) and Seps1 knockdown on myogenic differentiation, cells were differentiated for 72 h prior to transfection, and then 48 h posttransfection, myotubes were treated with differentiation media containing 0.35 mmol/L palmitate or vehicle as described above.

### Immunoblotting of C2C12 myoblasts and myotubes

Cells were lysed in M‐PER extraction buffer (Thermo Fisher Scientific) in the presence of protease inhibitors (Thermo Fisher Scientific). Five *μ*g (Seps1 and glucose regulated protein (GRP) 94) or 10 *μ*g (GRP78 and heme oxygenase‐1(HO‐1)) of total protein was separated on 4–20% acrylamide gradient gels (NuSep), and transferred to PVDF membranes. After blocking, membranes were incubated overnight with anti‐Seps1 (an in‐house rabbit polyclonal antibody against the COOH terminus of Seps1 generated as described here (Gao et al. 2003, 2004, 2007)), anti‐GRP94 (Cell Signaling, Danvers, USA; #2104), anti‐GRP78 (Cell Signaling; #3183), or anti‐HO‐1 (Assay Designs, Ann Arbor, USA; #SPA‐894) antibodies. This was followed by an incubation with a goat anti‐rabbit HRP‐linked secondary antibody (Cell Signaling; #7074). Bands were detected using ECL chemiluminescence (Invitrogen) and analyzed using Quantity One^®^ software (BioRad, Gladsville, AUS). The gels were stained with SimplyBlue™ SafeStain (Invitrogen) to validate even loading and protein expression was normalized to the optical density of the total protein per lane.

### RNA extraction and cDNA synthesis

Extraction and purification of RNA was performed using TRIzol reagent (Invitrogen) and the RNeasy^®^ Mini kit (Qiagen). RNA was quantitated using the Agilent Bioanalyzer and RNA 6000 Nano Assay kit (Agilent Technologies, Mulgrave, AUS). Complementary DNA was synthesized using the SuperScript™ III First‐Strand Synthesis System (Invitrogen).

### Real‐time PCR

Gene expression levels were determined by real‐time PCR using Brilliant^®^ SYBR^®^ Green QPCR Master Mix (Agilent Technologies), with PCR conditions were as follows: 95°C for 10 min (one cycle); 95°C for 30 sec and 60°C for 1 min (40 cycles). Relative gene expression was calculated as 2^−∆Ct^ following normalization to cyclophillin, a housekeeping gene. See Table 1 for forward and reverse primer sequences.

**Table 1:** Primer sequences for real‐time PCR

Gene	Forward primer (5′‐3′)	Reverse primer (5′‐3′)
*Seps1*	accgagagcctgcgattc	ggatgacaatgtagagtaggatgc
*Grp78*	ttcctgcgtcggtgtattca	gcggttgccctgatcgt
*Grp94*	gtatgtacgccgcgtattgatc	tcggaatccacaacacctttg
*Chop*	gtccctagcttggctgacaga	tggagagcgagggctttg
Ho‐1 (*Hmox1*)	gccaccaaggaggtacacat	gcttgttgcgctctatctcc
*Txnip*	atcgtggcgtggcaagag	cgtagatcagcaaggagtattcg
*Trx1*	cgtggtggacttctctgctacgtggtg	ggtcggtatgcatttgacttcacagtc

### Cell viability following Seps1 gene suppression and palmitate treatment

The mitochondrial reduction of MTT (3‐[4,5‐dimethylthiazol‐2‐yl]‐2,5‐ diphenyltetrazolium bromide; thiazolyl blue; Sigma‐Aldrich) was used to assess myotube viability (Mosmann 1983).

Exclusion of *7*‐aminoactinomycin D (7‐AAD; Sigma‐Aldrich) was used to assess myoblast viability. Following transfection and palmitate treatment, adherent and detached myoblasts were collected and fixed in 1% formaldehyde containing 2% FCS and 2 *μ*g/mL Actinomycin D. Cells were stored at 4°C in the dark until analysis on a FACS Calibur^®^ system with Cell Quest Software (Becton Dickinson Biosciences, North Ryde, AUS) to determine the proportion of dead and dying cells.

### Cell cycle progression following seps1 gene suppression

To discriminate between the G_0_, G_1_, S, and G_2_/M phases of the cell cycle, myoblasts were stained with fluorescein isothiocyanate (FITC; Thermo Fisher Scientific) for cellular protein and propidium iodide (PI) for DNA (Boquest et al. 1999). C2C12 myoblasts were trypsinized and fixed in ethanol for 6 h at 4°C, then stained overnight at 4°C with 50 *μ*g/*μ*L PI and 0.1 *μ*g/mL FITC plus 20 *μ*g/mL RNase in PBS followed by flow cytometry analysis using the FACS Calibur system and Cell Quest Software.

### Oxidative stress assays

To assess the effects of palmitate and/or Seps1 knockdown on cellular ROS, myotubes were incubated with 10 *μ*mol/L 5‐(and‐6)‐carboxy‐2′,7′‐dichlorodihydrofluorescein diacetate (DCFDA; Thermo Fisher Scientific). DCFDA oxidation was detected using a FlexStationII^384^ fluorometer (Molecular Devices; excitation = 485 nm and emission = 538 nm). H_2_O_2_ (25 *μ*mol/L) was used as a positive control.

Amplex^®^ Red (10‐acetyl‐3,7‐dihydroxyphenoxazine; 50 *μ*mol/L; Thermo Fisher Scientific) and 0.1 U/mL of horseradish peroxidase (HRP) were used to measure H_2_O_2_ production in muscle cells following Seps1 knockdown and/or palmitate treatment. Resorufin, the reaction product of Amplex^®^ Red oxidation, was detected using a FlexStation II^384^ fluorometer (Molecular Devices; excitation = 570 nm and emission = 585 nm). Glucose oxidase (25 mU/ml) was used as a positive control. The increase in absorbance (FU) was normalized to the protein content of each well and presented as a fold‐change increase in H_2_O_2_.

Reduced (GSH) and oxidized (GSSG) glutathione levels were measured, as previously described (Conlan et al. 2010), to determine the GSH:GSSG ratio in myotubes following 24 h of palmitate treatment or in myoblasts following Seps1 gene suppression.

### Statistics

All values are expressed as mean ± SEM. Results were analyzed using a 2‐way GLM‐ANOVA with the factors being siRNA and palmitate treatment, and where appropriate a Tukey's post hoc test. For the differentiation time course experiment, results were compared with a one‐way ANOVA and a Tukey's post hoc test. A *P* ≤ 0.05 was considered statistically significant.

## Results

### Seps1 expression profile in differentiating and palmitate‐treated muscle cells

**Figure 1:**
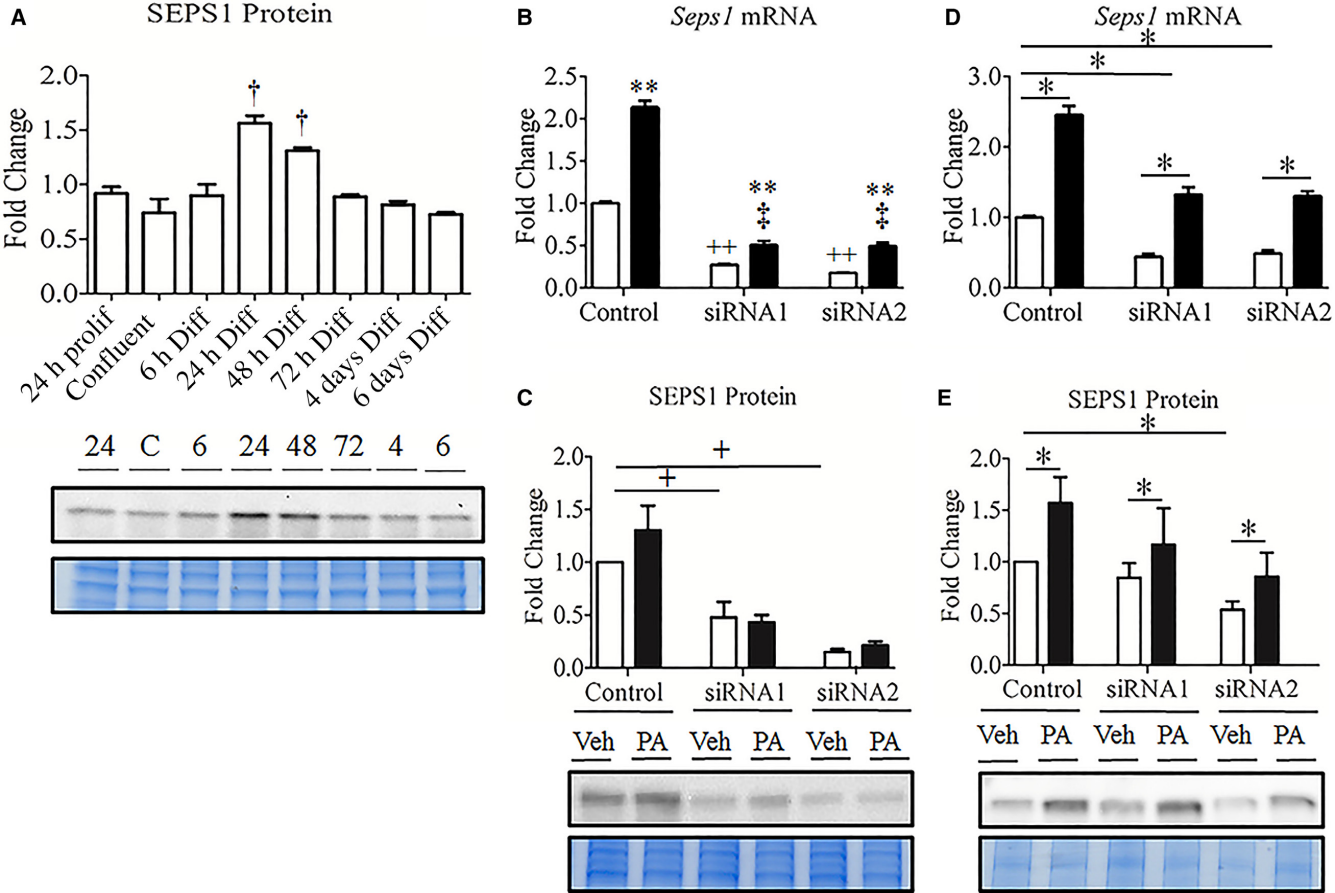
Seps1 expression in differentiating, palmitate‐ and siRNA‐treated C2C12 muscle cells. Cells treated with 0.1 mmol/L (myoblasts) or 0.35 mmol/L (myotubes) palmitate (black bars) or the corresponding ethanol/BSA vehicle (white bars). (A) Seps1 protein expression was assessed in proliferating and differentiating myoblasts, with a transient increase observed between 24 h and 48 h postdifferentiation when compared to proliferating myoblasts. C2C12 myoblasts were transiently transfected with one of two Seps1 siRNA constructs or a scramble control construct prior to treatment with 0.1 mmol/L (palmitate; black bars) or ethanol/BSA vehicle (vehicle; white bars) for 21 h. To assess gene knockdown and the effect of palmitate (B) *Seps1* mRNA and (C) protein levels were determined. C2C12 myotubes were also transiently transfected with one of two Seps1 siRNA constructs or a scramble control construct prior to treatment with 0.35 mmol/L (palmitate; black bars) or ethanol/BSA vehicle (vehicle; white bars) for 24 h. Followed by measurement of (D) *Seps1* gene and (D) protein expression. ^†^ *P *<* *0.0001 compared to proliferating myoblasts (one‐way GLM‐ANOVA). **P <* *0.05 for vehicle compared to palmitate‐treated cells (interaction; two‐way GLM‐ANOVA). ^++^ *P *<* *0.05 for Seps1 siRNA compared to scramble control following vehicle treatment (interaction; two‐way GLM‐ANOVA). ^‡^ *P *<* *0.05 for Seps1 siRNA compared to scramble control following palmitate treatment (interaction; two‐way GLM‐ANOVA). *P <* *0.05 compared to vehicle‐treated cells (main effect treatment; two‐way GLM‐ANOVA). ^+^ *P *<* *0.05 compared to scramble control (main effect siRNA; two‐way GLM‐ANOVA). For the myoblast differentiation and myotube siRNA experiments, cells were seeded in duplicate with three independent biological replicates and for the myoblast siRNA experiments, cells were seeded in triplicate with two independent biological replicates.

To first assess basal Seps1 protein levels in proliferating myoblasts and myotubes, a differentiation time‐course was performed where myoblasts, once confluent, were differentiated for up to 6 days. Seps1 protein expression was transiently increased up to twofold at 24 h and 48 h postdifferentiation (*P *<* *0.001; Fig. 1A), but was not sustained revealing similar expression levels in myoblasts and myotubes at 72 h to 6 days postdifferentiation. Thus, the myoblast and myotube cell culture models used in this study appeared to have similar basal Seps1 protein levels prior to gene knockdown.

The effect of palmitate on Seps1 expression, and whether two siRNAs designed to target *Seps1* mRNA were able to knockdown Seps1 in both myoblasts and myotubes, was investigated next. In myoblasts, 0.1 mM palmitate increased *Seps1* mRNA transcript abundance by 2.5‐fold, irrespective of gene knockdown (*P *<* *0.001; Fig. 1B). Nonetheless, in vehicle‐ and palmitate‐treated myoblasts, both *Seps1* siRNA constructs suppressed mRNA levels by approximately 75% (*P *<* *0.001; Fig. 1B). A reduction in Seps1 protein expression by both siRNAs was also measured in myoblasts with a 50% and 75% decrease following treatment with siRNA1 and siRNA2, respectively (*P *<* *0.001; Fig. 1C). Palmitate treatment (0.1 mmol/L), however, did not significantly increase Seps1 protein expression under any conditions tested (Fig. 1C).

In myotubes, 0.35 mmol/L treatment with palmitate increased Seps1 mRNA twofold regardless of siRNA treatment (*P *<* *0.001; Fig. 1D), and also significantly increased Seps1 protein levels (*P *<* *0.05; Fig. 1E). While siRNA treatment achieved approximately 50% knockdown of *Seps1* mRNA with both siRNA1 and siRNA2 (*P *<* *0.001; Fig. 1D), significant suppression of protein levels by 50% was only observed with siRNA2 (*P *<* *0.001; Fig. 1E). It is worth noting that this decrease in Seps1 protein expression achieved with siRNA2 in vehicle‐ and palmitate‐treated myotubes is similar to the protein knockdown achieved in myoblasts transfected with the siRNA1 construct.

### Reduced viability and proliferation following Seps1 knockdown in palmitate‐treated muscle cells

**Figure 2:**
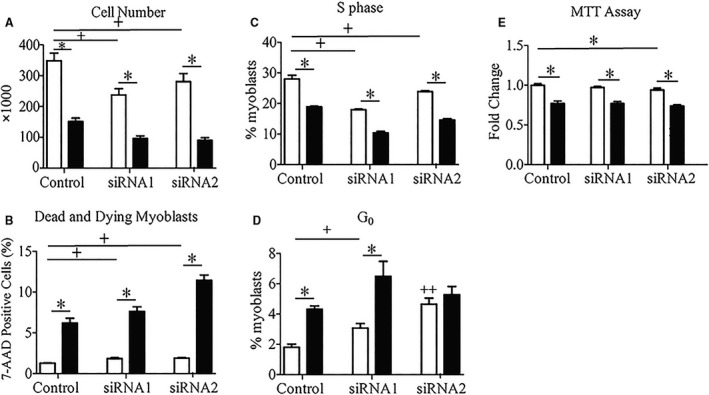
Cell cycle state and viability of C2C12 muscle following knockdown of Seps1 in the presence of palmitate. Cells treated with 0.1 mmol/L (myoblasts) or 0.35 mmol/L (myotubes) palmitate (black bars) or the corresponding ethanol/BSA vehicle (white bars). To assess the effects of Seps1 knockdown and nutrient stress on myoblast proliferation and differentiation, (A) manual cell counts were completed and (B) the percentage of dead and dying cells measured using 7‐AAD incorporation. FITC and PI staining were used to assess cell cycle state; specifically, (C) the proportion of myoblasts in S phase (DNA synthesis) and (D) G_0_ (quiescence). (E) The mitochondrial oxidation of MTT was used to assess the viability of myotubes following Seps1 siRNA and/or palmitate treatment. ^+^ *P *<* *0.05 compared to scramble control (main effect siRNA; two‐way GLM‐ANOVA). *P <* *0.05 compared to vehicle‐treated cells (main effect treatment; two‐way GLM‐ANOVA). ^++^ *P *<* *0.05 for Seps1 siRNA compared to scramble control following vehicle treatment (interaction; two‐way GLM‐ANOVA). To determine cell number and the proportion of dead or dying myoblasts, cells were analyzed in quadruplicate with three independent biological replicates. To assess cell cycle progression, myoblasts were analyzed in quadruplicate with three independent biological replicates. For the MTT assay in myotubes, an *N *= 8 wells and two independent biological replicates were analyzed.

To investigate how Seps1 modulates cell viability in response to lipid stress, the effect of Seps1 knockdown in both myoblasts and myotubes was assessed. Myoblast number was reduced in all palmitate‐treated cells (*P *<* *0.001). Whilst, Seps1 protein levels were not significantly increased in response 0.1 mmol/L palmitate treatment, Seps1 does modulate myoblast viability and senescence. Seps1 knockdown in myoblasts revealed a decrease in cell number up to 30% or 40% in vehicle‐ or palmitate‐treated myoblasts, respectively (*P *<* *0.001; Fig. 2A). This was attributed to greater cell death and decreased proliferation. The proportion of dead and dying myoblasts was increased in myoblasts transfected with either siRNA1 or siRNA2 (*P *<* *0.001; Fig. 2B). Furthermore, the proportion of proliferating myoblasts in the S phase (DNA synthesis) of the cell cycle was decreased in cells transfected with both siRNA constructs following vehicle or palmitate treatment (*P *<* *0.001; Fig. 2C). To complement this, the proportion of quiescent myoblasts in the G_0_ phase of the cell cycle was increased in vehicle‐ and palmitate‐treated myoblasts transfected with siRNA1 (*P *=* *0.005; Fig. 2D). Treatment with siRNA2 also increased the proportion of cells in the G_0_ phase, even in the absence of palmitate (*P *<* *0.02; Fig. 2D). Thus, even in the absence of lipid stress, Seps1 suppression was sufficient to reduce proliferation and promote cell cycle exit. Indeed, there is evidence in the literature linking reduced selenoprotein levels, including Seps1, to replicative senescence (Legrain et al. 2014).

In myotubes, following transfection with the siRNA2 construct, a trend toward a marginal (3–5%) decrease in viability was observed in vehicle‐ or palmitate‐treated myotubes (*P *=* *0.05; Fig. 2E), with no effect on cell viability observed in the siRNA1 transfected myotubes. As expected, palmitate also decreased myotube viability (*P* < 0.001; Fig. 2E).

### Seps1 gene suppression is associated with increased ROS levels in myoblasts and decreased ROS levels in myotubes

**Figure 3:**
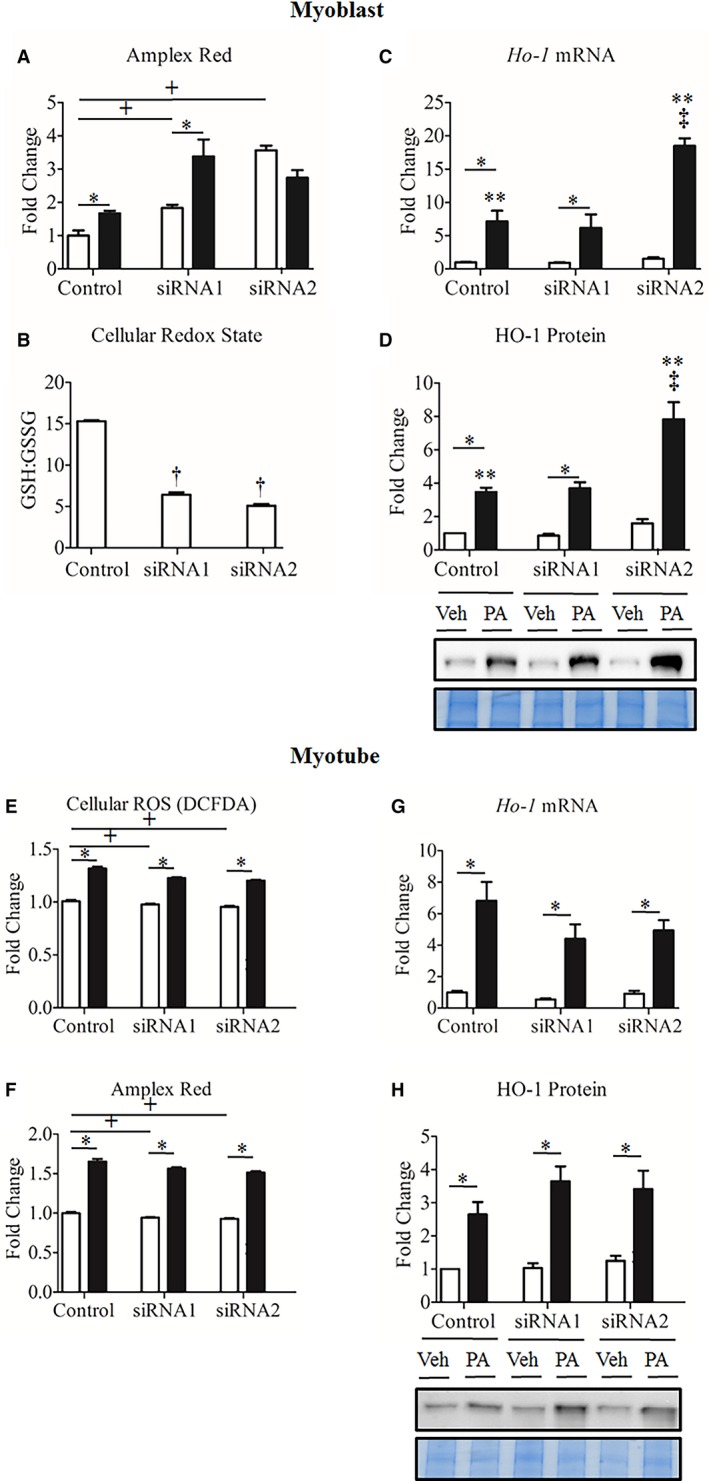
Differential oxidative stress responses in palmitate‐treated C2C12 myoblasts and myotubes following Seps1 knockdown indicate the importance of cell phenotype. Myoblasts treated with 0.1 mmol/L palmitate (black bars) or ethanol/BSA vehicle (white bars) or myotubes treated with 0.35 mmol/L palmitate (black bars) or ethanol/BSA vehicle (white bars). To assess the antioxidant properties of Seps1 in proliferating myoblasts, (A) H_2_O_2_ levels via Amplex^®^ Red, (B) cellular redox state via the GSH:GSSG ratio and (C) *Ho‐1* gene and (D) protein expression were measured in palmitate‐ and vehicle‐treated myoblasts transfected with Seps1 siRNA or scramble control constructs. To assess the antioxidant properties of Seps1 in differentiated myoblasts, (E) cellular ROS levels via the oxidation of DCFDA, (F) H_2_O_2_ via the oxidation of Amplex^®^ Red assay, (G) *Ho‐1* gene and (H) protein expression were measured in palmitate and vehicle‐treated myotubes transfected with the Seps1 siRNA or scramble control constructs. ^+^ *P *<* *0.05 compared to scramble control (main effect treatment; two‐way GLM‐ANOVA). *P <* *0.05 compared to vehicle‐treated cells (main effect siRNA; two‐way GLM‐ANOVA). ^†^ *P *<* *0.05 compared to scramble control (independent T‐test). **P <* *0.05 for vehicle compared to palmitate‐treated cells (interaction; two‐way GLM‐ANOVA). ^‡^ *P *<* *0.05 for Seps1 siRNA compared to scramble control following palmitate treatment (interaction; two‐way GLM‐ANOVA). In myoblasts, for the Amplex Red assay cells were seeded in quadruplicate with three independent biological replicates; to assess the GSH:GSSG ratio cells were seeded in triplicate with two independent biological replicates; and to measure Ho‐1 gene and protein expression cells were seeded in triplicate with two independent biological replicates. In myotubes, to assess cellular ROS, cells were cultured in *N* = 7 wells with two independent biological replicates; for the Amplex Red assay, cells were cultured in quadruplicate with three independent biological replicates; and to measure Ho‐1 gene and protein expression cells were cultured in duplicate with three independent biological replicates.

In vehicle‐treated myoblasts, Seps1 knockdown alone increased H_2_O_2_ levels significantly for both siRNAs (*P *<* *0.001; Fig. 3A), while the addition of palmitate further increased H_2_O_2_ levels (*P *<* *0.001). Following on from these observations, cellular redox state was assessed, and even in the absence of lipid stress, Seps1 suppression was associated with a more oxidized GSH:GSSG ratio (*P *<* *0.001; Fig. 3B). *Ho‐1* expression, assessed as a marker of second phase antioxidant responses (Martin et al. 2004), revealed increased mRNA transcript abundance and protein levels in the presence of palmitate (*P *<* *0.001 and *P *<* *0.001, respectively; Fig. 3C–D). Treatment with siRNA2, but not siRNA1, exacerbated the upregulation of *Ho‐1* gene and protein expression in the presence of palmitate when compared to cells treated with the scramble siRNA construct (*P *<* *0.01; Fig. 3C–D).

In line with the observed decrease in myotube viability, palmitate increased ROS levels (*P *<* *0.001; Fig. 3E–F), and *Ho‐1* gene and protein expression (*P *<* *0.001; Fig. 3G–H). Unlike in myoblasts, Seps1 knockdown in myotubes did not increase oxidative stress in the basal state nor in response to the lipid stress. Instead, Seps1 knockdown reduced ROS (*P *<* *0.001; Fig. 3E) and H_2_O_2_ concentrations (*P *≤* *0.001; Fig. 3F). Furthermore, Seps1 knockdown had no significant effect on *Ho‐1* mRNA and protein levels (Fig. 3G–H).

### Seps1 gene suppression increased gene and protein markers of ER and oxidative stress in myoblasts but not in myotubes

**Figure 4:**
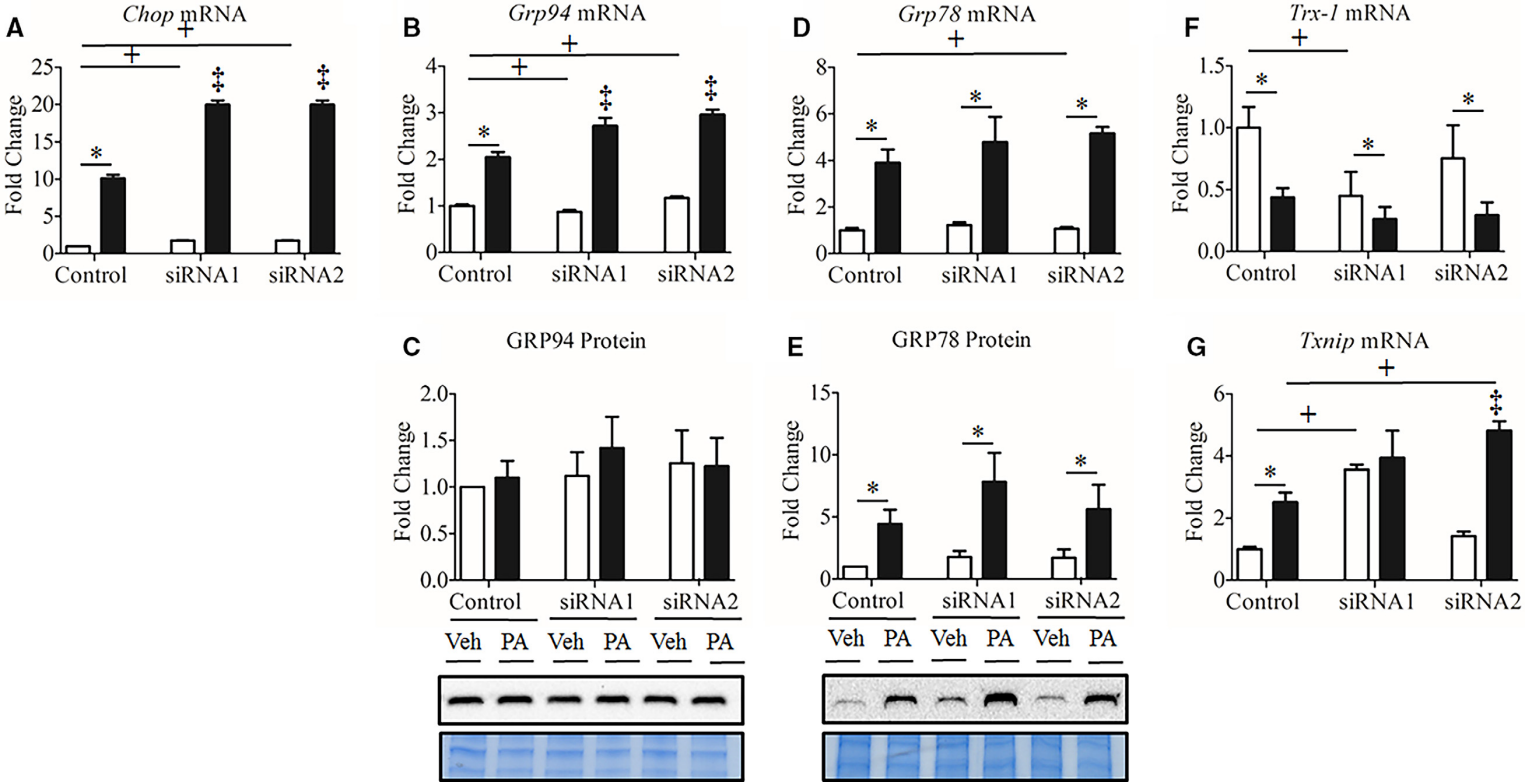
ER stress response in palmitate‐treated C2C12 myoblasts following Seps1 knockdown. Myoblasts treated with 0.35 mmol/L palmitate (black bars) or ethanol/BSA vehicle (white bars). To assess whether Seps1 is associated with the unfolded protein response and/or the thioredoxin antioxidant system, (A) *Chop* gene expression, (B) *Grp94* gene and (C) protein expression, (D) *Grp78* gene and (E) protein expression, (F) Thioredoxin‐1 (*Trx‐1*) gene expression, and (G) Thioredoxin inhibitor protein (*Txnip)* gene expression were measured in palmitate‐ and vehicle‐treated myoblasts transfected with the Seps1 siRNA or scramble control constructs. ^+^ *P *<* *0.05 compared to scramble control (main effect siRNA; two‐way GLM‐ANOVA). *P <* *0.05 compared to vehicle‐treated cells (main effect treatment; two‐way GLM‐ANOVA). ^‡^ *P *<* *0.05 for Seps1 siRNA compared to scramble control following palmitate treatment (interaction; two‐way GLM‐ANOVA). For gene and protein analysis, myoblasts were seeded in triplicate with two independent biological replicates.

Markers of ER stress response and the thioredoxin antioxidant system were also measured in myoblasts and myotubes. In myoblasts, Seps1 knockdown with either siRNA1 or siRNA2 exacerbated the upregulation of *Chop* (C/EBP homologous protein) and *Grp94* mRNA in the presence of palmitate (Fig. 4A–B). *Grp78* gene expression also tended to increase irrespective of vehicle or palmitate treatment with siRNA2 treatment (*P *=* *0.054; Fig. 4D). Despite increased *Grp94* and *Grp78* mRNA expression following Seps1 knockdown, their respective protein levels were not significantly altered (Fig. 4C–E). Given the role of the thioredoxin antioxidant system in regulating Seps1 oxidoreductase activity (Liu et al. 2013), metabolic stress (Kaimul et al. 2007), oxidative stress (reviewed in (Nishinaka et al. 2001)) and ER stress (Liu et al. 2015), the effects of palmitate and Seps1 knockdown on thioredoxin‐1 (*Trx‐1*) and its endogenous inhibitor thioredoxin binding protein (*Txnip*) were determined. *Trx‐1* gene expression was reduced in myoblasts transfected with the siRNA1, but not the siRNA2 construct, when compared to scramble control transfected myoblasts (*P *=* *0.019), with palmitate also decreasing *Trx‐1* mRNA levels (*P *<* *0.02; Fig. 4F). *Txnip* mRNA transcript abundance was increased in myoblasts transfected with the siRNA1 construct compared to scramble control transfected myoblasts, irrespective of palmitate or vehicle treatment (*P *<* *0.001; Fig. 4G). In myoblasts transfected with the siRNA2 or scramble control construct, palmitate increased *Txnip* mRNA transcript abundance (*P *<* *0.001; Fig. 4G). This increase was greater in palmitate‐treated myoblasts transfected with the siRNA2 construct versus the scramble control construct (*P *<* *0.001; Fig. 4G). Altogether these observations seem to indicate, that in proliferating myoblasts exposed to nutrient stress, Seps1 may provide greater protective effects against oxidative than ER stress, as *Grp78 and Grp94* expression increased only at the mRNA, but not protein level.

**Figure 5:**
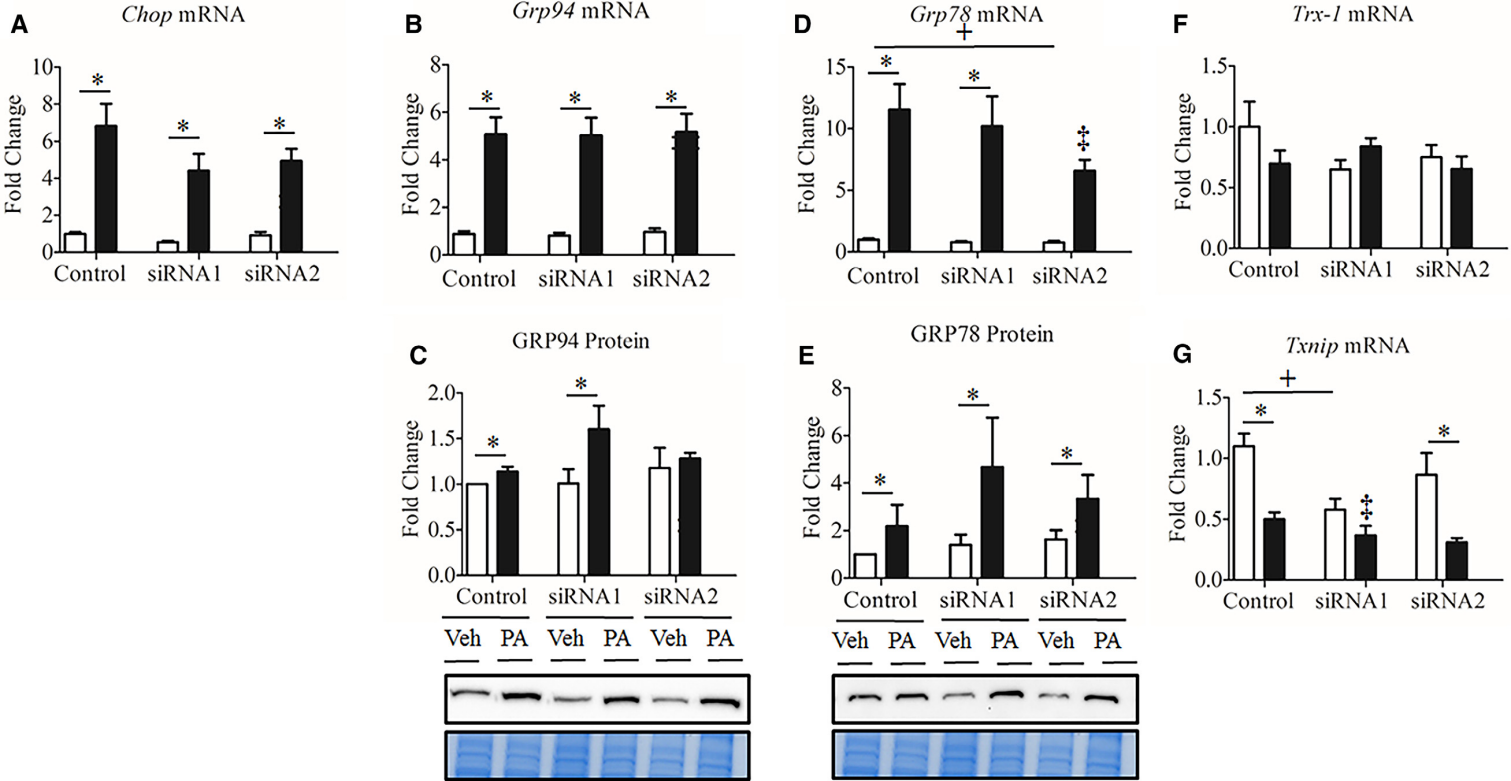
ER stress response in palmitate‐treated C2C12 myotubes following Seps1 knockdown. Myotubes treated with 0.35 mmol/L palmitate (black bars) or ethanol/BSA vehicle (white bars). To investigate whether cell cycle state or metabolic phenotype differentially affects the association between Seps1 and the unfolded protein response or the thioredoxin antioxidant system, (A) *Chop* gene expression, (B) *Grp94* gene and (C) protein expression, (D) *Grp78* gene and (E) protein expression, (F) Thioredoxin‐1 (*Trx‐1*) gene expression, and (G) Thioredoxin inhibitor protein (*Txnip)* gene expression were measured in palmitate‐ and vehicle‐treated myotubes transfected with the Seps1 siRNA or scramble constructs. *P <* *0.05 compared to vehicle‐treated cells (main effect treatment; two‐way GLM‐ANOVA). ^+^ *P *<* *0.05 compared to scramble control (main effect siRNA; two‐way GLM‐ANOVA). ‡*P* < 0.05 for cells treated with siRNA1 and palmitate compared to siRNA1 vehicle control (interaction; two‐way GLM‐ANOVA). For gene and protein analysis, myotubes were grown in duplicate with three independent biological replicates.

**Figure 6:**
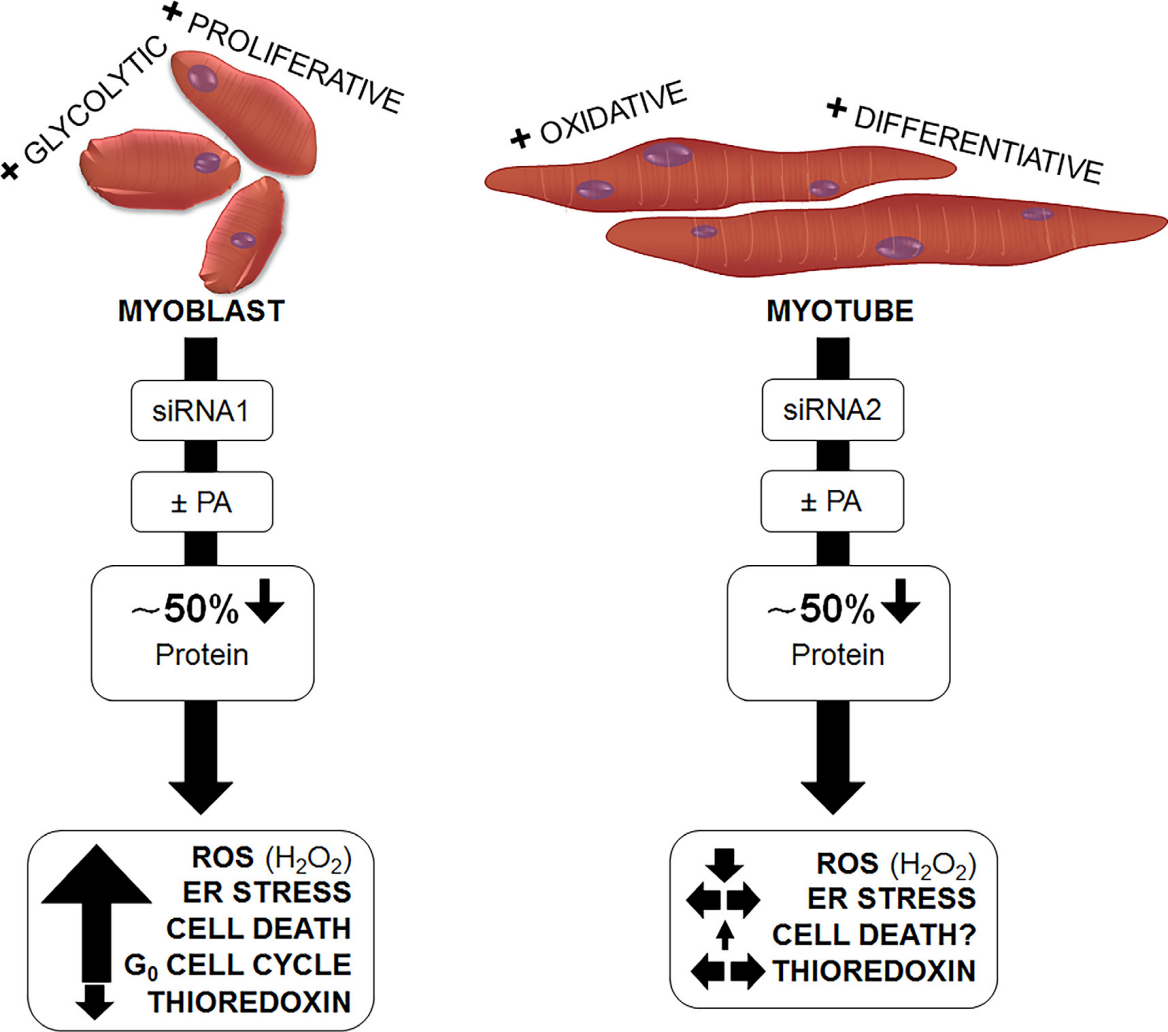
Seps1 protects the glycolytic proliferative myoblasts from lipid‐induced cell death. The genetic reduction of Seps1 by siRNA1 or siRNA2 in myoblasts and myotubes, respectively results in a 50% reduction in Seps1 protein expression. In the more glycolytic myoblast, reduction of Seps1 causes increased ROS and ER stress, leading to increased cell death and cell cycle exit (quiescence). However in the more oxidative myotubes, Seps1 reduction reduced ROS and has mild change in ER stress and cell death. Thus, the reduction of Seps1 affects the more glycolytic myoblasts, suggesting Seps1 may protect muscle cells of a glycolytic phenotype.

In myotubes, as expected, gene markers of ER stress, *Chop, Grp78* and *Grp94*, were increased at either or both mRNA and protein levels in response to palmitate treatment (*P *<* *0.001; Fig. 5A–E); however, no additional increase in their expression was observed following Seps1 knockdown. In contrast to observations in myoblasts, the mRNA transcript abundance of *Trx‐1* was unaffected by Seps1 suppression or palmitate treatment (Fig. 5F). While, Seps1 knockdown with siRNA1 or siRNA2 construct was associated with decreased *Txnip* gene expression (*P *<* *0.001 and *P *=* *0.056, respectively; Fig. 5G). Palmitate also decreased *Txnip* mRNA transcript abundance (*P *<* *0.001; Fig. 5G). This is in contrast to our observations in myoblasts, where Seps1 gene knockdown and palmitate treatment increased *Txnip* expression (Fig. 4G). Altogether, these data demonstrate that Seps1 has distinct effects on oxidative and ER stress responses and on gene markers of the thioredoxin antioxidant system in proliferating myoblasts versus differentiated myotubes (Fig. 6).

## Discussion

In skeletal muscle, the sensitive regulation of cellular stress responses is essential for optimal metabolic and contractile function (Ji 2015; Bohnert et al. 2018). The importance of selenoproteins, in particular glutathione peroxidase and thioredoxin reductase, in protection from and adaptation to cellular stress is well recognized (Reeves and Hoffmann 2009). The biological function of the seven ER‐resident selenoproteins, which include Seps1, is less well described. However, there is increasing interest in this group of selenoproteins due to their role in regulating oxidative and ER stress responses and intracellular calcium homeostasis (Arbogast and Ferreiro 2010; Shchedrina et al. 2011; Yao et al. 2013; Pitts and Hoffmann 2017; Addinsall et al. 2018a). Here, we report for the first time that in skeletal muscle cells, that Seps1 expression is upregulated in response to nutrient stress from the saturated fatty acid palmitate. Furthermore, in response to Seps1 gene knockdown, cellular stress responses to not only palmitate, but also vehicle treatment, were increased in proliferating myoblasts; however, this was not observed in differentiated myotubes. When compared to myotubes, proliferating myoblasts have a more glycolytic phenotype (Wagatsuma and Sakuma 2013; Ryall et al. 2015), fewer mitochondria (Leary et al. 1998; Elkalaf et al. 2013), and are more susceptible to cellular stress (Szczesny et al. 2013; Oláh et al. 2015). Therefore, our findings suggest that the cell cycle state, metabolic phenotype, or a combination of both affects the regulation of cellular stress responses by Seps1 (Fig. 6). This notion that Seps1 plays a role in modulating cellular stress responses in proliferating cells is further supported with increased ROS levels following Seps1 gene knockdown observed with other in vitro studies using primary vascular smooth muscle cells (Ye et al. 2016), HUVEC endothelial cells (Zhao et al. 2013), and porcine kidney epithelial 15 (PK15) cells (Gan et al. 2017); cells that still have proliferative capacity. Alternatively, given that Seps1 knockdown in myotubes did not increase ROS levels, may suggest an alternative or perhaps redundant role for Seps1 in differentiated myotubes compared with observations made in proliferating myoblasts.

In myotubes, ER stress was increased in response to palmitate, but not Seps1 knockdown. As indicated by increased *Chop, Grp97,* and *Grp78* gene expression and increased GRP78 protein expression in all palmitate treated myotubes. Whereas in myoblasts, Seps1 knockdown, irrespective of palmitate treatment, increased *Chop, Grp97,* and *Grp78* gene, but not protein, expression. GRP78 is considered a master regulator of ER stress signaling pathways leading to the activation of the UPR to attenuate translation, increase the degradation of misfolded protein via ERAD, and to further upregulate the expression of ER chaperones (e.g., GRP78 and GRP94), or to apoptosis via CHOP (Zhu and Lee 2015). Unlike GRP78, GRP94 has a more selective role in ER stress responses and facilitates the folding and/or assembly of specific membrane associated and secreted proteins, and it also functions as a low affinity, high capacity calcium binding protein (Marzec et al. 2012). The lack of an increase in Grp94 and Grp78 protein content, despite increased mRNA transcript abundance, in response to Seps1 knockdown perhaps suggests a secondary or redundant role for Seps1 in regulating ER stress responses in proliferating myoblasts. Indeed, in Hepa1‐6 mouse hepatoma cells, Seps1 gene suppression also did not increase the protein expression of grp78 and Chop. Xbp1 protein levels, which are important in the UPR and protecting cells from apoptosis, were also not increased (Li et al. 2018). However, aside from ERAD (Shchedrina et al. 2011), the cellular mechanisms by which Seps1 modulates ER stress responses and cell viability are still under investigation. Recently, in differentiated 3T3‐L1 adipocytes, Seps1 knockdown reduced cell viability due to impaired signaling through IRE‐1*α* leading reduced Xbp1 expression. This in turn increased Jnk phosphorylation leading to an imbalance of Bcl2 family members and increased apoptosis (Men et al. 2018). To more comprehensively understand the potential role of Seps1 in regulating ER stress responses and myoblast viability, the IRE‐1*α* and Xnp1 axis should be interrogated further. Especially, given the observed interactions between Seps1 suppression and palmitate treatment and that palmitate is known to induce phospho‐Jnk (Gorgani‐Firuzjaee et al. 2014).

Cross‐talk between the ER and mitochondria is also important in regulating cellular homeostasis (Romagnoli et al. 2007; Marchi et al. 2014). In response to cellular stress, as part of an adaptive response, mitochondria can buffer the excess calcium released from the ER. Emerging evidence suggests that increased mitochondrial content and respiratory capacity can promote survival from ER stress (Knupp et al. 2018). Seps1 has recently been shown to modulate ER calcium homeostasis and mitochondrial calcium buffering in Hepa1‐6, which then compromised cell viability via mitochondrial dysfunction (Li et al. 2018). Perhaps, the greater mitochondrial content and oxidative capacity of myotubes versus myoblasts (Leary et al. 1998; Elkalaf et al. 2013) attenuates the importance of Seps1 in protecting against cellular stress.

Seps1 is most potent as an antioxidant when coupled with thioredoxin, which is known to regulate various stress responsive signaling pathways including NF‐*κ*B. Together, they have an important role in maintaining cell viability and homeostasis (Matthews et al. 1992; Sakurai et al. 2004; Liu et al. 2013). Thioredoxin is inhibited by TXNIP and increased TXNIP expression is associated with impaired cell viability and metabolic dysfunction (Chen et al. 2008; Alhawiti et al. 2017). Furthermore, there is evidence linking TXNIP with ER stress and inflammation (Liu et al. 2015; Szpigel et al. 2017). In proliferating myoblasts, Seps1 suppression and/or palmitate treatment decreased the mRNA transcript abundance of *Trx‐1*, whilst increasing *Txnip* gene expression. Whereas in myotubes, Seps1 suppression and/or palmitate treatment decreased *Txnip* mRNA transcript abundance, with no change in *Trx‐1* gene expression. Perhaps, this differential regulation of *Txnip* and *Trx‐1* in response to excess lipid, also contributes to the observed increase in oxidative stress and associated reduction in cell viability in myoblasts, but not myotubes, following Seps1 knockdown. This study highlights a novel association found between Seps1, *Trx‐1* and *Txnip,* and our data lend support to earlier biochemical studies demonstrating a functional association between Seps1 and thioredoxin (Liu et al. 2013). Given the relevance of these proteins to metabolic disease, this association warrants further mechanistic and in vivo investigation.

The idea that glycolytic cells might be more reliant on Seps1 is in concordance with observations in dystrophic *mdx* mice, where the genetic reduction of Seps1 exacerbated the inflammatory profile of fast, glycolytic *extensor digitorum longus* (EDL), but not slow, oxidative *soleus* muscles (Wright et al. 2017). As an end point marker of heightened cellular stress, Seps1 reduction decreased myofiber cross‐sectional area in EDL, but not soleus, muscles (Wright et al. 2017). Furthermore, in Seps1 global knockout mice on a C57BL/6 background, the genetic reduction (by 50%) or deletion of Seps1 was associated with impaired contractile function, specifically force producing capacity, in fast phenotype EDL, but not slow phenotype *soleus* muscles (Addinsall et al. 2018b). To date, the majority of cell culture studies investigating Seps1 function, with the exception of preliminary work in adipocytes, have not accounted for cell cycle state or metabolic phenotype. Adipogenic differentiation of cultured 3T3‐L1 cells depends on the degradation of Seps1 by dexamethasone. In these cells, this resultant increase in ER stress is necessary for adipocyte maturation in vitro (Kim and Kim 2013). The observations that the cell cycle state and/or metabolic phenotype affect how Seps1 modulates cellular stress responses to excess lipid have interesting biological implications. For example, maternal obesity is associated with increased circulating lipid levels (including palmitate) and is a risk factor for impaired skeletal muscle development and metabolic dysfunction (Du et al. 2010a). Fetuses from obese ewes demonstrate impaired myogenesis and reduced myofiber growth (Du et al. 2010a), with reduced myoblast proliferation being a likely contributing factor (Zhu et al. 2004). As another example, vascular smooth muscle cells can transition between a proliferating or differentiated, contractile phenotype depending on biological context or disease pathology (Rzucidlo et al. 2007). Seps1 is highly expressed in blood vessels, in particular in vascular smooth muscle (Ye et al. 2016), and genetic studies of *SEPS1* gene sequence polymorphisms have revealed an association with cardiovascular disease (Cox et al. 2013; Yu et al. 2016). Similar to our observations in C2C12 myoblasts, in proliferating rat primary VSMCs, Seps1 gene knockdown increased cellular susceptibility to oxidative and ER stress and was associated with activation of stress responsive signaling pathways (Ye et al. 2016). Whether Seps1 modulates cellular stress responses in differentiated VSMCs has to date not been determined.

In summary, we have shown that Seps1 gene and protein expression is upregulated in cultured muscle cells in response to excess palmitate. However, the protection against lipid‐induced cellular stress afforded by Seps1 is more significant in proliferating, glycolytic myoblasts compared to differentiated, more oxidative myotubes, where the effect of Seps1 suppression was relatively minor in myotubes irrespective of palmitate treatment. The functional significance and underlying mechanism of how Seps1 regulates cellular stress responses to nutrient stress in muscle cells, especially those with different metabolic and proliferative capacities potentially has interesting implications for metabolic and musculoskeletal disease.

## Conflict of Interest

No conflict of interest to report.
